# Myelin Basic Protein as a Novel Genetic Risk Factor in Rheumatoid Arthritis—A Genome-Wide Study Combined with Immunological Analyses

**DOI:** 10.1371/journal.pone.0020457

**Published:** 2011-06-03

**Authors:** Chikashi Terao, Koichiro Ohmura, Masaki Katayama, Meiko Takahashi, Miki Kokubo, Gora Diop, Yoshinobu Toda, Natsuki Yamamoto, Human Disease Genomics Working Group, Rheumatoid Arthritis (RA) Clinical and Genetic Study Consortium, Reiko Shinkura, Masakazu Shimizu, Ivo Gut, Simon Heath, Inga Melchers, Toshiaki Manabe, Mark Lathrop, Tsuneyo Mimori, Ryo Yamada, Fumihiko Matsuda

**Affiliations:** 1 Center for Genomic Medicine, Graduate School of Medicine, Kyoto University, Kyoto, Japan; 2 Department of Rheumatology and Clinical Immunology, Graduate School of Medicine, Kyoto University, Kyoto, Japan; 3 Global Centers of Excellence (COE) program, Kyoto University Graduate School of Medicine, Kyoto, Japan; 4 Core Research of Evolutional Science and Technology (CREST) program, Japan Science and Technology Agency, Kawaguchi, Saitama, Japan; 5 Center for Anatomical Studies, Graduate School of Medicine, Kyoto University, Kyoto, Japan; 6 Department of Immunology and Genomic Medicine, Kyoto University Graduate School of Medicine, Kyoto, Japan; 7 Commisariat a l'energie Atomique (CEA), Institut Genomique, Centre National de Genotypage, Evry, France; 8 Clinical Research Unit for Rheumatology, University Medical Center, Freiburg, Germany; 9 Laboratory of Diagnostic Pathology, Graduate School of Medicine, Kyoto University, Kyoto, Japan; 10 Fondation Jean Dausset, Centre d'Etude du Polymorphisme Humain, Paris, France; 11 Unit of Statistical Genetics, Center for Genomic Medicine Graduate School of Medicine Kyoto University, Kyoto, Japan; 12 Institut National de la Sante et de la Recherche Medicale (INSERM) Unite U852, Kyoto University Graduate School of Medicine, Kyoto, Japan; Ohio State University Medical Center, United States of America

## Abstract

Rheumatoid arthritis (RA) is a major cause of adult chronic inflammatory arthritis and a typical complex trait. Although several genetic determinants have been identified, they account for only a part of the genetic susceptibility. We conducted a genome-wide association study of RA in Japanese using 225,079 SNPs genotyped in 990 cases and 1,236 controls from two independent collections (658 cases and 934 controls in collection1; 332 cases and 302 controls in collection2), followed by replication studies in two additional collections (874 cases and 855 controls in collection3; 1,264 cases and 948 controls in collection4). SNPs showing *p*<0.005 in the first two collections and *p*<10^−4^ by meta-analysis were further genotyped in the latter two collections. A novel risk variant, rs2000811, in intron2 of the myelin basic protein (*MBP*) at chromosome 18q23 showed strong association with RA (*p* = 2.7×10^−8^, OR 1.23, 95% CI: 1.14–1.32). The transcription of *MBP* was significantly elevated with the risk allele compared to the alternative allele (*p*<0.001). We also established by immunohistochemistry that MBP was expressed in the synovial lining layer of RA patients, the main target of inflammation in the disease. Circulating autoantibody against MBP derived from human brain was quantified by ELISA between patients with RA, other connective tissue diseases and healthy controls. As a result, the titer of anti-MBP antibody was markedly higher in plasma of RA patients compared to healthy controls (*p*<0.001) and patients with other connective tissue disorders (*p*<0.001). ELISA experiment using citrullinated recombinant MBP revealed that a large fraction of anti-MBP antibody in RA patients recognized citrullinated MBP. This is the first report of a genetic study in RA implicating MBP as a potential autoantigen and its involvement in pathogenesis of the disease.

## Introduction

Rheumatoid arthritis (RA) is the most common cause of adult inflammatory arthritis, affecting 0.5–1% of the adult population worldwide, and is associated with joint pain, dysfunction and deformity. Both genetic and environmental risk factors have been implicated in RA [1]–[2]. *HLA-DRB1* is a major genetic component of RA across ethnicities and is estimated to contribute to 30 to 50% of the total genetic risk [3]. However, the other risk loci identified to date show ethnic-specific patterns of disease association. Large-scale genetic analyses including genome-wide association (GWA) studies have shown that more than 20 genes such as *PTPN22*, *TRAF1/C5*, *CD40*, and *TNFAIP3* are associated with RA in populations of European descent [4]–[12]. A different set of non-*HLA* genes, namely, *PADI4*, *SLC22A4*, *FCRL3*, *CD244* and *CCR6* were first reported for their association with RA using Japanese DNA collections [13]–[17]. Among them, several genes including *CCR6*, *STAT4* and *TNFAIP3* were later proven their association beyond ethnicity [12], [18]–[19]. On the other hand, some other genes showed strong specificity to a certain ethnic group. The association of the *PTPN22* has been repeatedly reproduced by subsequent genetic studies in Europeans [5], [20]–[21]. However, no evidence of strong disease risk in *PTPN22* was shown in Japanese in part due to a much lower frequency of the risk allele [22]. Similarly the association of *PADI4* with RA, which has been confirmed by multiple genetic studies in Japanese and Koreans [23]–[24], is found to be much weaker in Europeans [25]–[26]. Moreover, the size of DNA collections used for GWA studies is much larger in European populations than in Japanese, suggesting the existence of unknown genetic factors in Japanese [12], [17]. For these reasons, we decided to conduct a new large-scale GWA study of RA in Japanese. Independent collections of RA patients and controls were enrolled from four clinical centers in our study. The collections from two centers, totaling 990 cases and 1,241 controls, were characterized with genome-wide SNP arrays, and the data were analyzed to identify potential disease-associated loci. For replication, SNPs at these loci were examined in the two remaining collections, totaling 2,138 cases and 1,803 controls.

## Results

### Genome scan and validation studies

We accumulated data on 3,128 cases and 3,039 controls of four independent RA collections (termed as collections 1,2,3 and 4, Table S1). Collections 1 and 2 (totaling 990 cases and 1,236 controls) were used for GWA analysis and collections 3 and 4 (totaling 2,138 cases and 1,803 controls) were used as replication samples. Quality control of the GWA genotyping results was undertaken separately in cohorts 1 and 2 because of differences in the SNP arrays used (see Methods, Table S2). For 225,079 markers that were common between the arrays and fulfilled our inclusion criteria, we found no evidence of population stratification between cases and controls (genomic control inflation factor λ = 1.03, Figure S1). We undertook analysis of each collection individually, and a meta-analyses to pool the results in the two collections in the association analysis (see Methods for further details). We report *p*-values from the meta-analysis unless otherwise stated.

We found a strong association of disease risk with markers in the *HLA* complex [27] (*p* = 5.0×10^−31^, Table S3). Although no other chromosomal loci showed genome-wide significance, we detected evidence of association signal in *PADI4* (*p* = 2.3×10^−5^), as previously reported in Japanese [13], [23], [27] (Table S3). The association results of five reported genes in Japanese were shown in Table S4. These results support the quality of our study populations for genetic analysis. In addition, we identified 10 SNPs in five additional chromosomal regions that met our statistical criteria for testing in the replication collections (*p*<0.005 in both collections 1 and 2, and *p*<10^−4^ in the meta-analysis). None of them showed potential association with *p*-value being smaller than 10^−5^ in the other Japanese GWA study [17]. From each of these regions, the SNP with the smallest *p*-value was selected for examination in collections 3 and 4. One of these SNPs, rs2000811 (*p* = 0.0036 in collection1; *p* = 5.7×10^−4^ in collection2; *p* = 1.2×10^−5^ in the meta-analysis) located on chromosome 18q23, was significantly associated with RA in both replication collections (*p* = 0.023 in collection3; *p* = 0.0041 in collection4, and *p* = 3.0×10^−4^ in collections 3 and 4). When the four collections were combined, the evidence of association at rs2000811 exceeded genome-wide significance when evaluated either by meta-analysis (*p* = 2.7×10^−8^; OR = 1.23; 95%CI 1.14–1.32) or by pooling of the genotype counts (*p* = 4.0×10^−8^; OR = 1.23; 95%CI 1.14–1.32; see Table 1). There was no difference in the effect size among the four collections (*p* = 0.28). To be more conservative, however, we have calculated corrected *p*-values with Principle Component Analysis (PCA), using subsets of case and control collections for which individual genotypes were available (970 cases and 297 controls, for details, see Materials and Methods). There was no difference in *p*-values with and without the correction (*p* = 6.5×10^−4^ and corrected *p* = 6.5×10^−4^). The four SNPs from the other regions that were tested showed no evidence of association in collection3 for replication study (Table S5).

**Table 1:** Association of *MBP* locus with rheumatoid arthritis in the Japanese population.

Chr	dbSNPID	Gene	Allele	DNA Collection		Genotype counts			Success rate	HWE*p*	RAF**	*p*-value	OR	*mhp* ***
			Ref.(A1)/Var.(A2)			A1A1	A1A2	A2A2					(95%CI)	
18q23	rs2000811	*MBP*	C/T*	1	case	203	303	136	99.8	0.25	0.45	0.0036	1.25	
					control	344	442	148	100	0.76	0.4		(1.08–1.44)	
				2	case	95	152	79	99.7	0.24	0.48	5.7×10^−4^	1.49	
					control	120	131	46	100	0.31	0.38		(1.19–1.87)	
				3	case	283	392	182	98.1	0.034	0.44	0.023	1.17	
					control	298	404	134	97.8	0.88	0.4		(1.02–1.34)	
				4	case	393	622	233	98.7	0.63	0.44	0.0041	1.19	
					control	341	451	141	98.4	0.68	0.39		(1.06–1.35)	
				3+4	case	676	1014	415	98.5	0.32	0.44	3.0×10^−4^	1.18	
					control	639	855	275	98.1	0.69	0.4		(1.08–1.30)	
				pooled	case	974	1469	630	98.9	0.078	0.44	4.0×10^−8^	1.23	2.7×10^−8^
					control	1103	1428	469	98.9	0.85	0.39		(1.14–1.32)	

*risk allele for the disease,

**risk allele frequency, and

***p-value in meta-analysis using Cochran-Mantel-Haenszel test.

**Figure 1:**
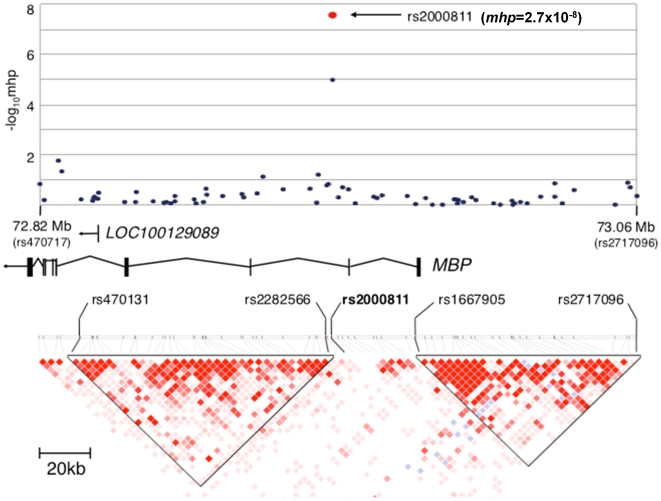
A schematic view of the association results and LD structure of the human *MBP* gene locus at chromosome 18q23. SNPs located between rs470131 and rs2717096 are plotted in −log_10_ scale according to their chromosomal positions and *p*-values calculated with Cochran-Mantel-Haenszel test. Red circle indicates *mhp*-value of rs2000811 by meta-analysis using the combined results of collections 1 to 4. Relative locations of the genes in the region are shown with their transcriptional orientations by arrows. LD blocks were generated using the genome scan results.

The disease associated marker rs2000811 is located in the second intron of the *MBP* (myelin basic protein) gene at chromosome 18q23 within a 156-kb region that contains the *MBP* gene (NCBI MapViewer, build 36.3). Linkage disequilibrium (LD) was evaluated using genotyping results obtained in collections 1 and 2; rs2000811 did not show significant LD with other markers from the region (r^2^<0.14; Figure 1), or elsewhere in the genome. An imputation analysis using the Japanese HapMap data identified a SNP, rs9958028, which was 358-bp apart and in strong LD with rs2000811 (r^2^ = 0.96), as the second strongest association. However, no other marker was in strong LD with these two markers (r^2^ = 0.35 or smaller) (Figure S2). To determine if unidentified polymorphisms within *MBP* were in LD with rs2000811, we performed a sequencing of the exons and the promoter region of the *MBP* gene in 84 Japanese population control DNAs (Method S1). We identified 66 SNPs, 37 of which were not registered in dbSNP, and three of which were deleterious polymorphisms (Tables S6 and S7). Again, none of these polymorphisms was in strong LD with rs2000811 (r^2^ = 0.35 or smaller) (Figure S3). An imputation analysis using the genotyping results obtained by sequencing did not discover any other polymorphisms showing stronger association signals (*p*>0.0070) than that of rs2000811. Taken together, these data suggest that rs2000811 and/or one or more other as yet unidentified non-coding polymorphisms within or near *MBP* are responsible for the genetic association.

### Evaluation of MBP transcription

**Figure 2:**
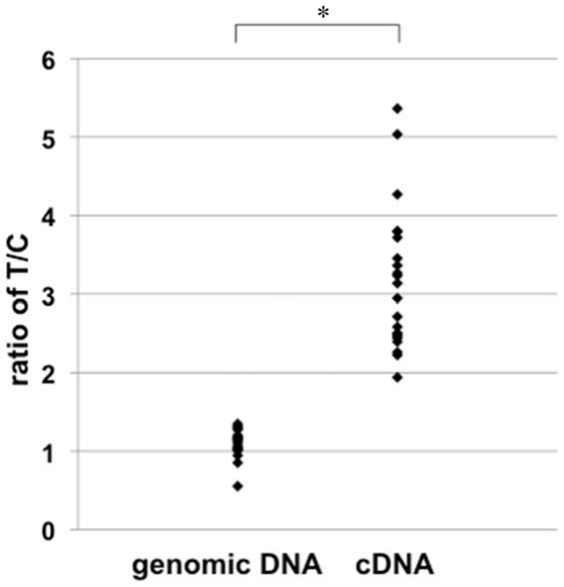
Allelic difference in *MBP* transcription using allele specific quantitative RT-PCR. The amount of *MBP* primary transcripts transcribed from chromosomes carrying rs2000811 risk (T) and alternative (C) alleles was compared in each cell line, and the ratio (T/C) was plotted. Genomic DNA was used as a control for equimoler biallelic representation. Experiments were done twice independently.

Quantitative RT-PCR experiments showed only very low levels of *MBP* expression in RNA from Epstein-Barr virus (EBV)-transformed human B-lymphoblastoid cell lines, and we could detect no discernable correlation of *MBP* transcript levels with different risk genotypes (*p* = 0.36, Figure S4). By a similar reason, *in-silico* expression analysis using GEO database did not return clear association [28]. However, when we performed allele-specific quantitative RT-PCR [16] using genomic DNA and cDNA of these cell lines, we observed elevated allele-specific transcription associated with the risk allele (*p*<0.001, Figure 2, for detailed procedure, see Materials and Methods). This suggests that rs2000811, and/or other variants in linkage disequilibrium with this marker, impact the quantitative pattern of *MBP* transcription. However, bioinformatics analysis identified no known *cis*-acting elements covering rs2000811 that could be inferred to have functional effects (Method S2). In addition, the alignment of the 4-kb region ranging between 2-kb centromeric and 2-kb telomeric to rs2000811 revealed that this segment has very low interspecies conservation among placental mammals.

### Expression of the MBP protein in synovial tissues

**Figure 3:**
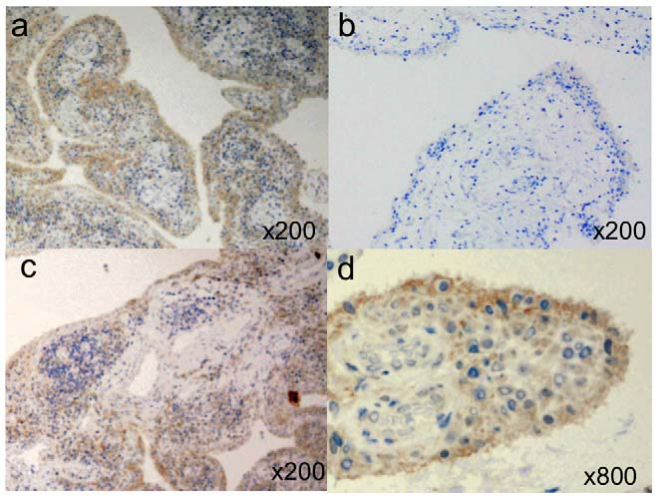
Immunohistochemistry of the MBP protein in human synovial tissues stained by monoclonal anti-MBP antibody. Synovial tissue of RA patients, in particular, along the synovial lining layer strongly expressed MBP (A), whereas that of non-inflammatory osteoarthritis patients was much weaker (B). The expression of MBP in the synovial lining layer was weaker near follicules of infiltrated lymphocytes (C). Localized expression of MBP was observed at the plasma membrane of synoviocytes (D).

Next we investigated the expression of the MBP protein in synovial tissue, as this is the main target of inflammation in RA. Microscopic observation revealed that MBP was highly expressed along the lining layer of synovial tissues in 20 out of 23 RA patients tested, while the expression of MBP was observed in only one out of five controls (*p* = 0.0017), and then generally at a weaker level (Figure 3A, B). In synovial tissue from RA patients, the detected MBP expression was weaker in synovial lining layer adjacent to the follicules of infiltrated lymphocytes (Figure 3C). In synoviocytes, the expression of MBP was mainly observed in the plasma membrane (Figure 3D).

### Quantification of antibodies to MBP in RA patients

**Figure 4:**
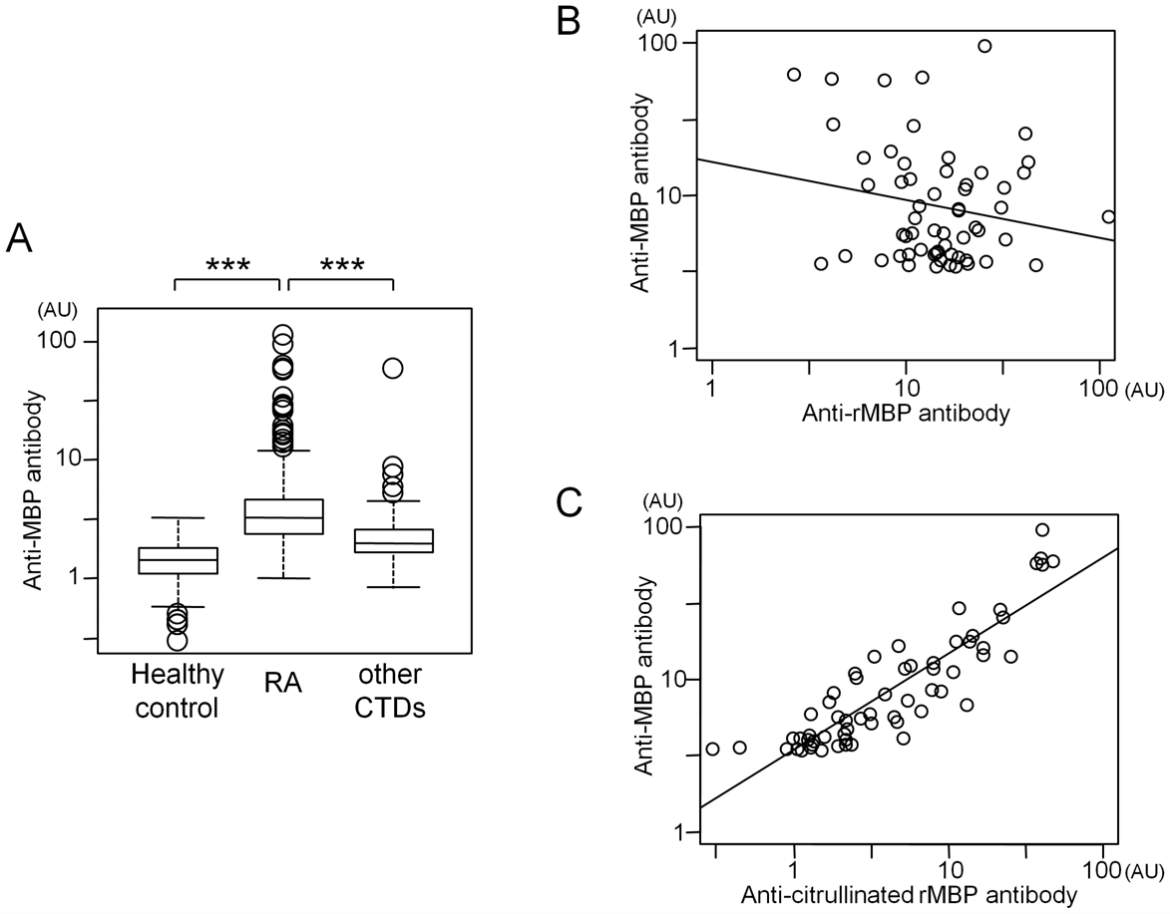
Quantification of circulating antibodies against MBP protein. A. Each boxplot indicates distribution of anti-MBP antibody titers in healthy controls, in RA patients, and in other connective tissue diseases (CTDs) (for detailed composition of the disease patients, see materials and methods). Results were representative of the two independent experiments. *** indicates statistical *p*-value smaller than 0.001. B. Correlation of autoantibody levels between human brain-derived MBP and recombinant MBP (rMBP). C. Correlation of autoantibody levels between human brain-derived MBP and citrullinated rMBP. Sixty RA patients who were positive for anti-MBP antibody were used for the analysis. In each figure, antibody titers were shown as arbitrary unit (AU).

Antibodies to MBP are the major component of autoantibodies in multiple sclerosis, a human autoimmunity with a neurodegenerative phenotype [29]. To assess a possible association of circulating antibodies to MBP with RA, we quantified these in plasma from 323 RA cases, 131 healthy controls and 162 patients with other connective tissue diseases (disease controls) by Enzyme-linked immunosorbent assay (ELISA) with MBP purified from human brain as antigen. The average levels of anti-MBP antibody in plasma of RA patients were much higher than those of healthy controls and patients with seven other connective tissue diseases (*p*<0.001; Figure 4A). Specificity in detection of anti-MBP antibody in ELISA experiments was confirmed by immunoblotting using plasma of a subset of RA patients and controls (Figure S5, Method S3). We also confirmed that the enhancement of ELISA signals by non-specific binding of IgG- and IgM-RF in patients' sera was negligible (for details, see Method S4 and Figure S6).

Amino acid analysis of the MBP protein derived from human brain showed that approximately 21% (citrulline/arginine = 6.0/23.1) of the arginine residues in MBP was citrullinated in human brain under physiological conditions (Method S5). It is possible that anti-MBP antibody recognizes and binds to citrullinated MBP protein. We then performed ELISA using recombinant MBP protein and compared the antibody titers with those of human brain-derived MBP. We could not observe correlation between the two results (r = −0.19, Figure 4B). However, when we used recombinant MBP protein artificially citrullinated *in-vitro*, the ELISA results showed strong correlation in titers of the autoantibody (r = 0.88, Figure 4C). From these results, we concluded that higher levels of anti-MBP antibody in RA patients than in healthy controls and in patients with other connective tissue diseases was attributed to autoantibodies binding to citrullinated MBP.

## Discussion

We conducted a large-scale GWA-based genetic study of RA in the Japanese population. A genome scan of 225,079 SNPs in two DNA collections of RA patients followed by replication in two additional collections led to identification of a novel risk variant, rs2000811 (*p* = 2.7×10^−8^, OR 1.23, 95% CI: 1.14–1.32), in the second intron of the *MBP* gene at chromosome 18q23. This is the second largest genome-wide association study to date in the Japanese population, and the first to have identified chromosome 18q23 as a potential susceptibility locus for RA. 18q23 was not reported as a candidate genetic locus in recent GWA study of RA in Japanese [17]. As summarized in Table S4, we confirmed in the current study the association of three out of seven genes reported to date whereas there was no evidence of association for the other four genes. A comprehensive genetic study by a meta-analysis using the results of different genome scans followed by the validation study with a large number of patients and controls will clarify relative contribution of each genetic locus to RA in the Japanese population. There are no GWA studies to date that indicate chromosome 18q23 as a potential genetic locus related to the susceptibility to RA in European populations [5]–[12]. By taking into account the detection power of the meta-analysis by Stahl *et al.*, it is natural to consider that the association of *MBP* with RA is limited to Japanese (and possibly other Asian populations).

The disease-associated marker rs2000811 is isolated in an 18-kb segment of the intron, and does not show evidence of significant LD with known markers in the region or neighboring genomic regions (Figure 1). The LD structure of the human *MBP* locus was very similar between Europeans and Japanese, and there was no clear LD block in the region encompassing rs2000811 in both populations (Figure S2). Sequencing of the DNAs of 84 healthy controls failed to identify other polymorphisms that were in strong LD with rs2000811 in the exons or the promoter region of the *MBP* gene. While bioinformatics analysis failed to identify any *cis*-acting elements encompassing rs2000811, allele-specific expression analysis demonstrated the elevated transcription level of *MBP* with the risk allele. This raises the possibility that *cis*-acting regulatory elements that encompass unknown genetic variations in strong LD with rs2000811.

A strong expression of the MBP protein was observed in synoviocytes of RA patients while expression was weaker in those of non-inflammatory controls. The fact that the expression of MBP was strongest in the synovial lining layer suggests the role of anti-MBP antibody as a trigger of inflammatory reactions through attacking synoviocytes. Although a study investigating citrullinated proteins in synoviums did not clearly identify MBP [30], MBP is possibly one of the unidentified subset of the citrullinated proteins. MBP expression was weaker in synoviocytes adjacent to the follicules of infiltrated lymphocyte. It would be of interest to examine whether MBP in synovium is citrullinated and such phenomena are correlated with the disease activity, in particular, at different stages of proliferative synovitis but data for such an investigation are not available in our study. In the absence of a quantitative assay of expression in synoviocytes, it was not possible to discern a correlation between the levels of *MBP* expression with the risk genotype in our experiments.

MBP is a well-known target autoantigen in multiple sclerosis (MS), a human neurodegenerative disease with an active destruction of myelin sheath [29]. The MBP protein has six different isoforms of which isoforms 1, 2, 3 and 4 with shorter open reading frames are expressed preferentially in the central nervous system (CNS). Isoforms 7 and 8 with higher molecular weights (also called Golli-MBP) are known to be expressed relatively ubiquitously including cells of the hematopoietic lineage [31]. The fact that RA patients rarely present CNS symptoms may suggest the involvement of Golli-MBP protein in the generation of anti-MBP antibody and expression in the synovium. An immunological study using relatively small numbers of plasma samples showed that anti-MBP antibody is present in 60% (or six out of ten) of RA patients [32]. To our knowledge, however, there is no study which quantitatively characterized anti-MBP antibody in RA patients as well as in patients of other connective tissue diseases and in healthy controls. On the other hand, ACPA which recognizes citrullinated proteins by peptidylarginine deiminase is considered as a specific and predictive marker for RA [13], [33]–[34]. Indeed, several studies showed the existence of antibodies to citrullinated MBP in RA patients [35]–[36]. However, the role of MBP and anti-MBP antibody in the pathogenesis of RA is yet to be elucidated. In the current study, we found that a part of brain-derived MBP was citrullinated in physiological conditions and that higher levels of anti-MBP antibody in RA patients can be attributed to MBP citrullination, although anti-MBP antibody is not in complete cross-reactivity with ACPA. We examined whether or not rs2000811 was predominantly associated with patients who were positive for ACPA. However, there was no statistical difference in allele frequency of rs2000811 between patients with and without ACPA (*p* = 0.40, data not shown).

We observed no correlation between the levels of anti-MBP antibody and genotypes at the risk locus. The repertoire of autoantibody is likely to depend on numerous factors such as the immunogenicity and extent of citrullination of the MBP protein. Also, a series of immunological reactions including antigen presentation in thymus and peripheral organs, activation of dendritic cells, T- and B-lymphocytes, and balance of Th1/Th2 and effector/regulatory T-lymphocytes affect antibody production. A possible explanation for the lack of correlation is that the disease-associated genotypes affect these factors in a way that impacts risk without directly influencing the quantitative values of anti-MBP antibody, which we find to be highly variable between RA patients that we have studied. Therefore, it will be of interest to compare the expression levels of *MBP* transcripts and MBP epitopes in synovial tissue, and between different genotypes and disease activity.

Taken together, this is the first genetic study which identified *MBP*, an autoantigen gene, to be associated with RA in Japanese. Transcription of *MBP* was increased with the risk allele of the associated SNP, rs2000811. Strong expression of the MBP protein was observed in the synovial tissues of the patients. Furthermore, significant increase of circulating autoantibodies against MBP protein was demonstrated in RA patients as compared to those with other connective tissue diseases, implicating its role as a disease-associated biomaker.

## Materials and Methods

### Ethics Statement

Written informed consent was obtained from all the participants at the institute of sample collection after being approved for genetic studies by the local ethical committee, namely, Kyoto University Graduate School and Faculty of Medicine, Ethics Committee, The Ethic Committee, Sagamihara National Hospital, NHO, Dohgo Spa Hospital Ethical Committee, University of Tokyo Medical Research Center Ethics Committee, Tokyo Women's Medical University Genome Ethics Committee, Ethics Committee of Tokyo Women's Medical University and Aichi Cancer Center Ethical Committee for human genome research.

### Study subjects

RA collections 1 to 4 consisted of 658 affected individuals and 934 controls, 332 and 302, 874 and 855, and 1,264 and 948, respectively (summarized in Table S1). The case subjects of collections 1, 2 and 3 were recruited at the rheumatology departments of Kyoto University Hospital, Dohgo Spa Hospital, Sagamihara National Hospital, and Tokyo University Hospital, and those for collection4 were from Tokyo Women's Medical University. All cases fulfilled the revised criteria (1987) of the American College of Rheumatology (ACR) for rheumatoid arthritis [37]. Genotype count data of the Japanese Single Nucleotide Polymorphism (JSNP) database [38] were used as controls for collection1. The control subjects for collection2 were from the Department of Ophthalmology and Visual Science at Kyoto University Hospital [39]. DNA samples of healthy Japanese volunteers in Pharma SNP Consortium [40] and in Aichi Cancer Center Hospital and Research Institute [41] were used for collections 3 and 4, respectively. Plasma of 323 RA patients and 162 patients of other connective tissue diseases (38 of SLE, 25 each of Sjögren's syndrome and systemic sclerosis, 20 each of Behçet's disease and mixed connective tissue disease, 19 of polymyositis/dermatomyositis and 15 of vasculitis) were obtained at Kyoto University Hospital, and those of 131 healthy controls were from Dohgo Spa Hospital.

### Genome-wide association analysis

Genome scan for collections 1 and 2 was performed using Infinium Technology (Illumina Inc., San Diego, CA). Case subjects of collection1 were genotyped with Human-Hap300 (version 1.0, 302,627 SNPs) or Human CNV370-Duo (version 1.0, 332,270 SNPs). For collection2, case and control subjects were genotyped on Human610-Quad (version 1.0, 577,348 SNPs), and HumanHap550 (version 3.0, 547,163 SNPs), respectively. Validation studies using collections 3 and 4 were performed using Taqman technology (Applied Biosystems Inc., Foster City, CA) according to the manufacturer's specifications.

### Quality control and statistical tests for case-control association

277,420 SNPs that were common among the four arrays described above were chosen for association study. Publicly available genotype counts from the JSNP project were used as control collection1. For this collection, detailed information such as individual genotypes and cluster plots are not disclosed. DNA samples with a call rate smaller than 0.90 (three in collection1 cases), showing high degree of kinship (PI_HAT>0.10 by PLINK [42], eleven in collection1 cases, one in collection2 cases, and four in collection2 controls) and with evidence of possible contamination (one in collection2 controls) were removed from statistical analyses. PCA was performed using the genome scan results of the remaining 644 cases of collection1, in addition to 331 cases and 297 controls of collection2. At this stage, five DNA samples (one in collection1 cases, and four in collection2 cases) that did not fall into the Japanese cluster were removed. Regarding the SNP markers, a total of 225,079 SNPs with call rate greater than 0.95 for both cases and controls and minor allele frequency greater than 0.05 either in case or in control of each collection were used for analysis. The *p*-value for rs2000811 corrected by PCA was calculated using the remaining 970 cases and 297 controls and compared with the *p*-value without correction.

The case-control association was examined independently for collections 1 and 2 with Cochran-Armitage trend test, followed by meta-analysis with Cochran-Mantel-Haenszel (CMH) test by combining the two collections. Population stratification in collections 1 and 2 was examined and corrected with Genomic Control [43]. The SNPs that showed *p*<0.005 in both collections and meta-analysis *p*-value smaller than 10^−4^ were selected as candidates for further evaluation. Among multiple SNPs in the same region that fulfilled the above criteria, the SNP with the smallest *p*-value in the meta-analysis was chosen for validation with collections 3 and 4. To be more specific, among the four SNPs in the *PLEKHK1* region on chromosome 10q21, namely rs3910172 (*p* = 4.4×10^−5^), rs6479805 (*p* = 5.4×10^−5^), rs10733769 (*p* = 5.8×10^−5^), and rs4147233 (*p* = 6.6×10^−5^), rs3910172 was selected for the replication study. Likewise, in the region of chromosome 10p14, rs2026628 (*p* = 1.6×10^−5^) was chosen over rs11253857 (*p* = 5.3×10^−5^), and rs687848 (*p* = 5.1×10^−5^) was chosen over rs587404 (*p* = 7.1×10^−5^) in the *MACF1* region on chromosome 1p31–32. SNPs in the *HLA* and *PADI4* loci were not included in validation studies. Haploview version 4.1 software [44] was used for LD evaluation, and MapViewer (build 36.3) [45] was used to identify the location and structure of the genes in the region.

### Quantification of allelic difference in gene expression in MBP transcription

Allele specific gene expression analysis was performed as previously described [16]. Briefly, human B-lymphoblastoid cell lines transformed by EBV were obtained from the Health Science Research Resources Bank of Japan (Osaka). Genomic DNA and total RNA were extracted by standard procedures from 22 cell lines heterozygous for rs2000811 alleles. The ratio of MBP primary transcripts (hnRNA) was quantified between the risk and wild-type alleles by TaqMan assay with SNP genotyping probes. Genomic DNA of a cell line homozygous for the wild-type allele (C) were mixed with DNA homozygous for the risk allele (T) at eight different molar ratios (2∶1, 3∶2, 1∶1, 2∶3, 1∶2, 1∶3, 1∶4, 1∶6) to draw a standard curve for the evaluation of RNA quantity.

### Immunohistochemistry

Synovial tissue specimens of 23 RA patients and five non-inflammatory controls were obtained from Department of Diagnostic Pathology and Department of Orthopaedic Surgery in Kyoto University Hospital. Paraffin-embedded tissues were prepared in a standard method and sectioned at a thickness of 3 µm. The section was mounted on a glass slide coated with 2% 3-aminopropyl triethoxy silane (Tokyo Kasei, Tokyo, Japan). Immunohistochemical staining of MBP was performed by using the standard avidin-biotin-peroxidase complex (ABC) method, as previously described [46]. The sections were incubated overnight at 4°C with an affinity-purified murine anti-human MBP monoclonal antibody (Leica Microsystems, Wetzlar, Germany) diluted at 1∶100 in PBS. The sections were then incubated with biotinylated horse anti-mouse IgG antibody (Vector Lab, Burlingame, CA) for 40 minutes, followed by incubation with peroxidase-conjugated streptavidin (Vector Lab, Burlingame, CA) at room temperature for 50 minutes. The coloring reaction was performed with 0.3 mg/ml diaminobenzidine and 0.003% H_2_O_2_ in 50 mM Tris-HCl (pH 7.6). Each section was counterstained with haematoxylin. Evaluation of MBP expression was performed by a blind test by two rheumatologists as well as a pathologist, and statistical significance in positivity was calculated with Fisher's exact test.

### Enzyme-linked immunosorbent assay (ELISA)

Microtiter plates (Nalge Nunc International K.K., Tokyo, Japan) were coated with 50 µl of MBP protein at 5 µg/ml extracted from human brain (Sigma, St. Louis, MO) or with recombinant human MBP (Genscript, Piscataway, NJ.) in 50 mM bicarbonate buffer (pH 9.6) and incubated at 4°C overnight. Citrullination of recombinant human MBP was done *in-vitro* by rabbit skeletal PAD (Sigma, St. Louis, MO) for 3 hours as previously described [47]. After wells were washed and coated with phosphate-buffered-saline (PBS) containing 2% bovine serum albumin (BSA), 50 µl of plasma samples diluted to 1∶150 with 2% BSA in PBS containing 5 U/ml heparin (Mochida Pharmaceutical Co., Ltd. Tokyo, Japan) were added and incubated at room temperature for 2 hours. 50 µl each of purified goat anti-human IgG polyclonal antibody conjugated to alkaline phosphate (Millipore. Billerica, MA) diluted to 1∶2000 was added and incubated at room temperature for 1.5 hours. 50 µl of BCIP/NBT substrate (Sigma, St. Louis, MO) in 2 µM MgCl_2_ was then added and incubated for 45 minutes in the dark. The optical density (OD) value at 405 nm was measured by a SpectraMax Plus^384^ Microplate Reader (Molecular Devices, Sunnyvale, CA). A standard curve was generated by serial dilution (1∶50, 1∶150, 1∶500, 1∶1500 and 1∶5000) of a plasma sample with a high titer of anti-MBP antibody. The titer of diluted standard sample in 1∶50 was set as 100 U for human brain-derived MBP. All samples were examined in duplicate except for negative control in which plasma was replaced by PBS with 2% BSA and was measured in quadruplicate. The specificity of ELISA results was confirmed by standard immunoblotting analysis using sera of 10 each of RA patients and controls.

### Statistical analysis of ELISA results

The titer of anti-MBP antibody between RA or its subgroups and controls was compared with Wilcoxon rank-sum test. The correlation between the titers of autoantibody was estimated with Pearson's correlation coefficient in logarithm scale. These statistics were performed in the R statistical system (http://www.R-project.org) and SPSS(ver18).

## Supporting Information

Figure S1 **QQ plot to show the observed and expected** ***p*** **-values of the combined genome scan results.** Vertical and horizontal axes indicate observed and expected *p*-values, respectively (A) and in logarithmic scale (B). The analysis using genomic control method showed no significant effect of population stratification (λ_GC_ = 1.03) between the case and control groups.
(TIF)

Figure S2 **Imputation analysis and LD structure of the human** ***MBP*** **locus.** Imputation was performed using the case genotypes of collections 1 and 2 and control genotypes of collection2. Individual genotypes of control population of collection1 (JSNP) were not available. Determination of LD structure was performed by using the GWAS results in this study and the HapMap results of Japanese and Caucasians (Hap JPT and Hap CEU, respectively).
(TIF)

Figure S3 **LD structure of the 156-kb region spanning the** ***MBP*** **gene.** LD plot was generated with Haploview using polymorphisms with reference allele frequencies between 0.05 and 0.95.
(TIF)

Figure S4 **Quantification of allelic difference in** ***MBP*** **transcription.** Human B-lymphoblastoid cell lines transformed by EBV were obtained from the Health Science Research Resources Bank of Japan (Osaka, Japan). Total RNA was extracted by standard procedures from the cell lines that were either homozygous for the wild-type allele (50 cell lines), heterozygous (50 cell lines) or homozygous for the risk allele (49 cell lines) of rs2000811. The amount of *MBP* cDNA in each cell line was measured and normalized to that of β-glucronidase using Taqman Gene Expression Assay (for MBP; Hs00921943-m1, for β-glucronidase; Hs99999908_m1, Applied Biosystems Inc., Foster City, CA) in GeneAmp 7500 Sequence Detection System. The comparative ΔΔCT method and Jonckheere-Terpstra test were used for the analysis.
(TIF)

Figure S5 **Immunoblotting of anti-MBP antibody.** Immunoblotting analysis was performed to confirm specific binding of circulating anti-MBP antibody. Lane 1, 2 to 6 and 7 were incubated with control plasma, plasma of patients, and rabbit polyclonal anti-human MBP antibody, respectively. The intensity was variable between RA patients whereas no signal was obtained in controls. Similar results were obtained using plasma of the other five RA patients and nine controls.
(TIF)

Figure S6 **Comparison of circulating anti-MBP antibody and IgG-RF.** We investigated whether the presence of RF in sera enhances the signal in ELISA through binding of IgG-RF to the constant region of anti-MBP antibody or through non-specific binding of IgM-RF to secondary antibody used in the experiments. Initially, correlation between IgG-RF and anti-MBP antibody titers was assessed to examine whether IgG-RF had any influence on anti-MBP antibody titers. 114 patients with RA and 13 other connective tissue disease patients for whom IgG-RF measurement was available were enrolled in this evaluation. As a result, no correlation was observed between the titers of anti-MBP antibody and IgG-RF (Spearman's rank-sum coefficient being 0.145 with *p*-value of 0.103). Subsequently, ELISA experiments using human IgM or IgG as target antigens were undertaken. Non-specific binding of secondary antibodies to human IgM compared to IgG was less than 1%.
(TIF)

Table S1 **Summary of the study populations used for the association analysis.** Abbreviations were as follows; ACPA: antibodies to citrullinated peptide antigens, RF: rheumatoid factor, SD: standard deviation, N/A, not available.
(DOC)

Table S2 **Summary of quality control for genome scan results.**
(DOC)

Table S3 **Association of** ***HLA*** **and** ***PADI4*** **loci with rheumatoid arthritis in the Japanese population.** *risk allele for the disease, **risk allele frequency, and ***p-value in meta-analysis using Cochran-Mantel-Haenszel test.
(DOC)

Table S4 **Association of previously reported non-** ***HLA*** **genes in the current study.** *p-value in meta-analysis using Cochran-Mantel-Haenszel test.
(DOC)

Table S5 **Replication results of the four regions.** The order of SNPs is in accordance with *mhp*-value. Chromosome and dbSNPID refer to NCBI build 36.3. *P*-values are calculated using the Cochran-Armitage trend test. *risk allele for the disease, **risk allele frequency, and ***OR, odds ratio with 95% confidence interval. ***p-value in meta-analysis using Cochran-Mantel-Haenszel test.
(DOC)

Table S6 **Oligonucleotide primers used for sequencing of the exons and the promoter region of the** ***MBP*** **gene.**
(DOC)

Table S7 **The list of genetic polymorphisms discovered by sequencing the exons and the promoter region of the** ***MBP*** **gene.** *Positions of polymorphisms are according to NCBI Refseq Build 36.3. Polymorphisms are located between position 1 and position 2.
(DOC)

Method S1 **Sequencing of the exons and the promoter region of the MBP gene.**
(DOC)

Method S2 **Bioinformatics analysis.**
(DOC)

Method S3 **Immunoblotting of anti-MBP antibody.**
(DOC)

Method S4 **Evaluation of non-specific binding of secondary antibodies.**
(DOC)

Method S5 **Amino acid analysis.**
(DOC)
