# Supplementary figures and images for: Myelin Basic Protein as a Novel Genetic Risk Factor in Rheumatoid Arthritis—A Genome-Wide Study Combined with Immunological Analyses
Source: PLoS One. 2011 Jun 3;6(6):e20457. doi: 10.1371/journal.pone.0020457 (PMC3108877; doi:10.1371/journal.pone.0020457)

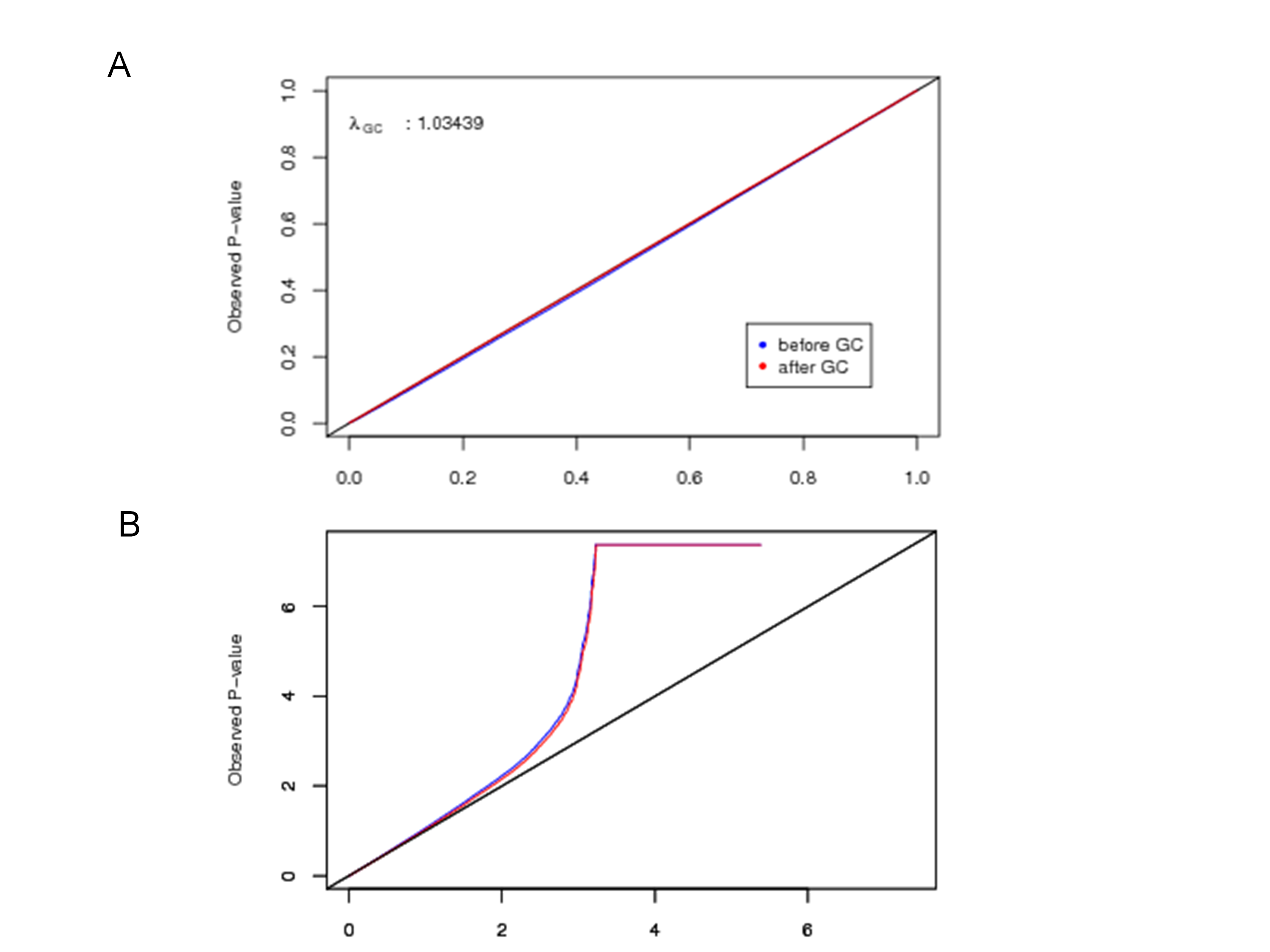

Supplement: Figure S1 — QQ plot to show the observed and expected p -values of the combined genome scan results. Vertical and horizontal axes indicate observed and expected p-values, respectively (A) and in logarithmic scale (B). The analysis using genomic control method showed no significant effect of population stratification (λGC = 1.03) between the case and control groups. (TIF) [file pone.0020457.s001.tif]

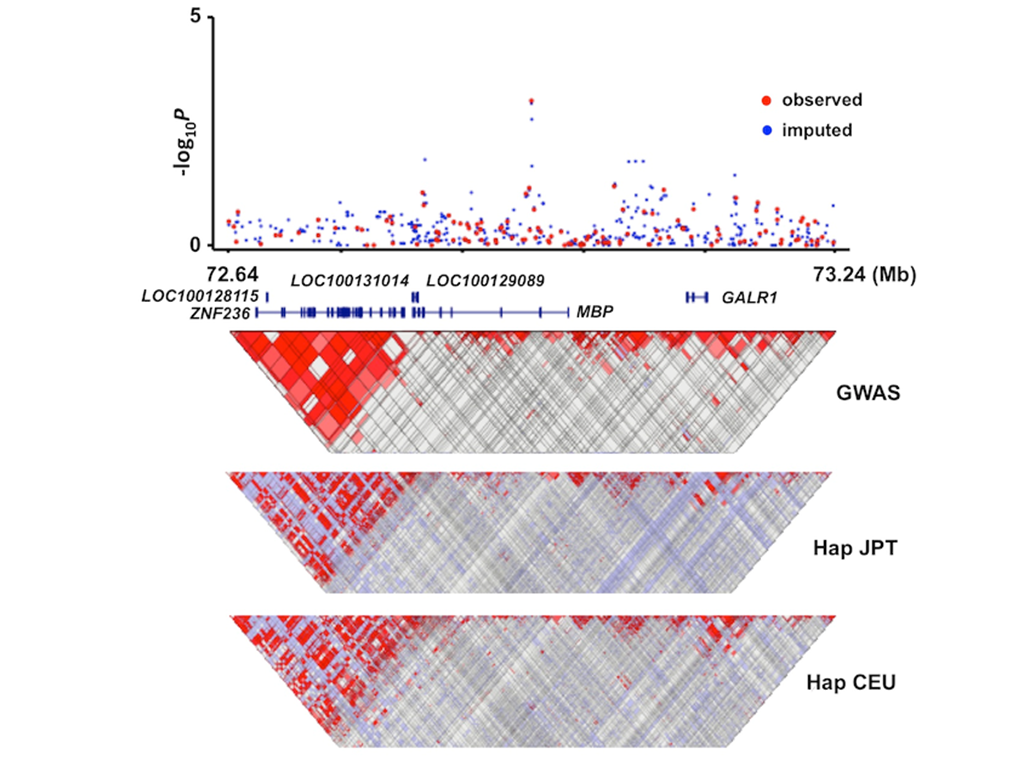

Supplement: Figure S2 — Imputation analysis and LD structure of the human MBP locus. Imputation was performed using the case genotypes of collections 1 and 2 and control genotypes of collection2. Individual genotypes of control population of collection1 (JSNP) were not available. Determination of LD structure was performed by using the GWAS results in this study and the HapMap results of Japanese and Caucasians (Hap JPT and Hap CEU, respectively). (TIF) [file pone.0020457.s002.tif]

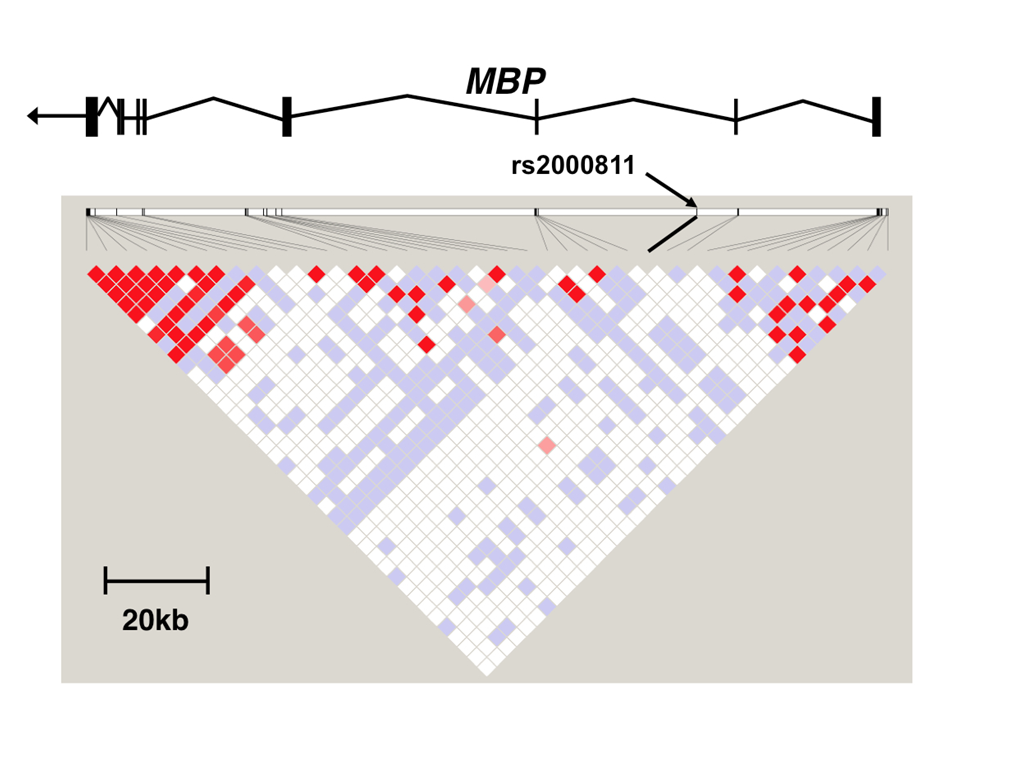

Supplement: Figure S3 — LD structure of the 156-kb region spanning the MBP gene. LD plot was generated with Haploview using polymorphisms with reference allele frequencies between 0.05 and 0.95. (TIF) [file pone.0020457.s003.tif]

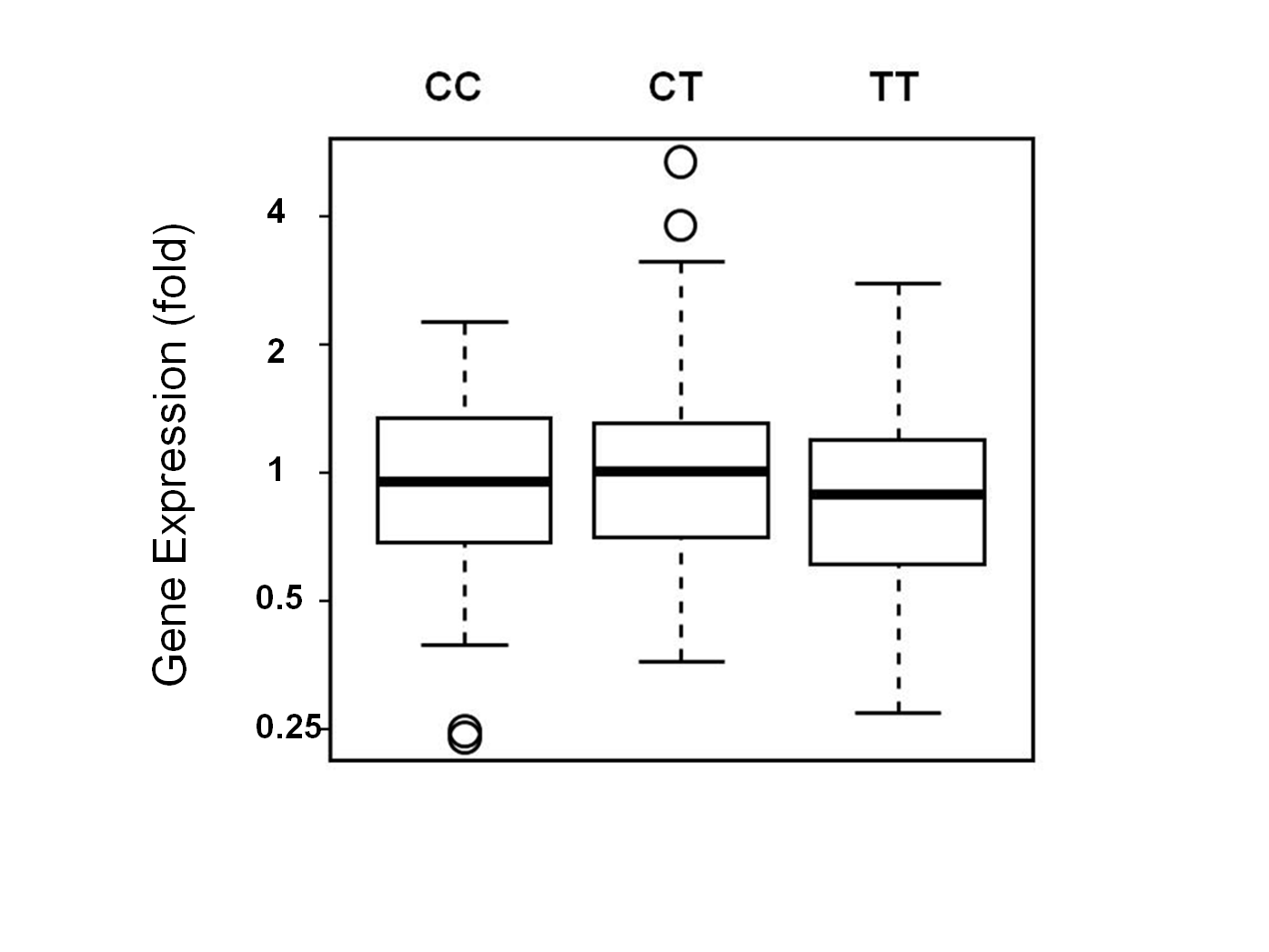

Supplement: Figure S4 — Quantification of allelic difference in MBP transcription. Human B-lymphoblastoid cell lines transformed by EBV were obtained from the Health Science Research Resources Bank of Japan (Osaka, Japan). Total RNA was extracted by standard procedures from the cell lines that were either homozygous for the wild-type allele (50 cell lines), heterozygous (50 cell lines) or homozygous for the risk allele (49 cell lines) of rs2000811. The amount of MBP cDNA in each cell line was measured and normalized to that of β-glucronidase using Taqman Gene Expression Assay (for MBP; Hs00921943-m1, for β-glucronidase; Hs99999908_m1, Applied Biosystems Inc., Foster City, CA) in GeneAmp 7500 Sequence Detection System. The comparative ΔΔCT method and Jonckheere-Terpstra test were used for the analysis. (TIF) [file pone.0020457.s004.tif]

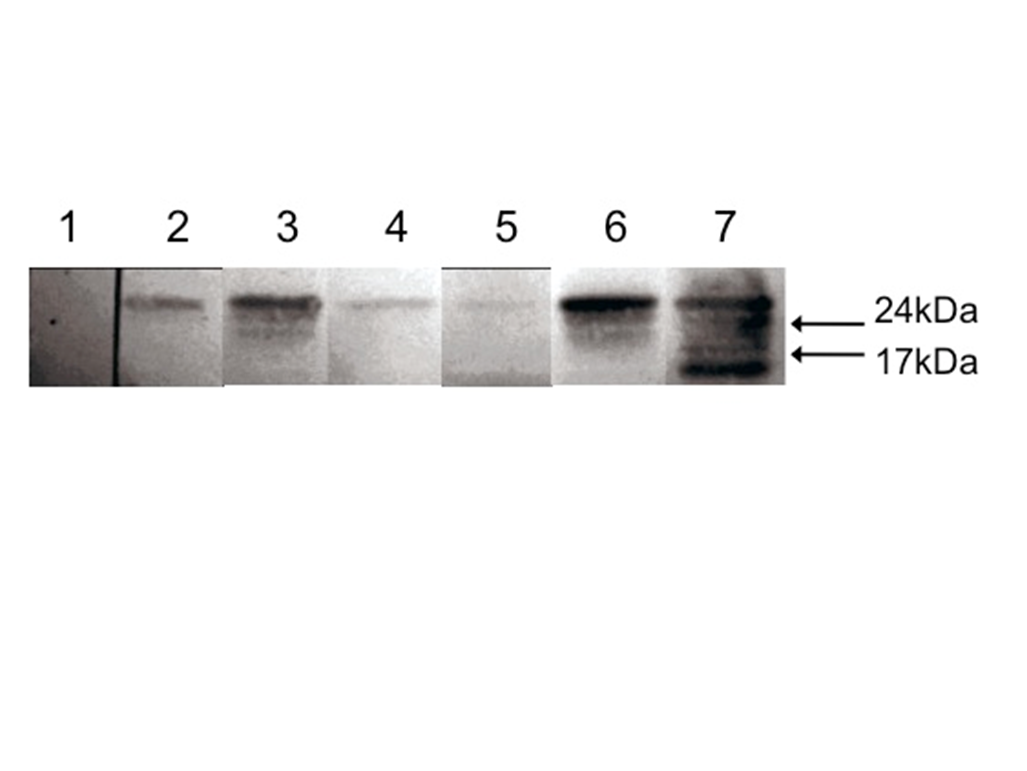

Supplement: Figure S5 — Immunoblotting of anti-MBP antibody. Immunoblotting analysis was performed to confirm specific binding of circulating anti-MBP antibody. Lane 1, 2 to 6 and 7 were incubated with control plasma, plasma of patients, and rabbit polyclonal anti-human MBP antibody, respectively. The intensity was variable between RA patients whereas no signal was obtained in controls. Similar results were obtained using plasma of the other five RA patients and nine controls. (TIF) [file pone.0020457.s005.tif]

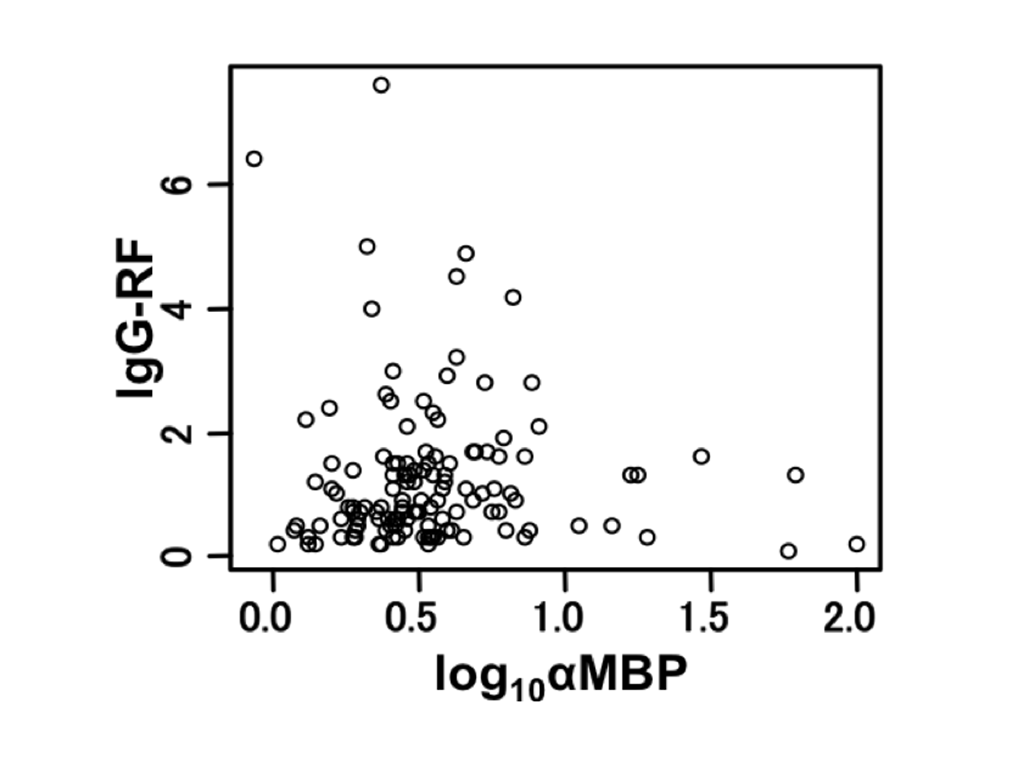

Supplement: Figure S6 — Comparison of circulating anti-MBP antibody and IgG-RF. We investigated whether the presence of RF in sera enhances the signal in ELISA through binding of IgG-RF to the constant region of anti-MBP antibody or through non-specific binding of IgM-RF to secondary antibody used in the experiments. Initially, correlation between IgG-RF and anti-MBP antibody titers was assessed to examine whether IgG-RF had any influence on anti-MBP antibody titers. 114 patients with RA and 13 other connective tissue disease patients for whom IgG-RF measurement was available were enrolled in this evaluation. As a result, no correlation was observed between the titers of anti-MBP antibody and IgG-RF (Spearman's rank-sum coefficient being 0.145 with p-value of 0.103). Subsequently, ELISA experiments using human IgM or IgG as target antigens were undertaken. Non-specific binding of secondary antibodies to human IgM compared to IgG was less than 1%. (TIF) [file pone.0020457.s006.tif]
