# Supplementary material for: Myelin Basic Protein as a Novel Genetic Risk Factor in Rheumatoid Arthritis—A Genome-Wide Study Combined with Immunological Analyses
Source: PLoS One. 2011 Jun 3;6(6):e20457. doi: 10.1371/journal.pone.0020457 (PMC3108877; doi:10.1371/journal.pone.0020457)
Supplement: Table S1 — Summary of the study populations used for the association analysis. Abbreviations were as follows; ACPA: antibodies to citrullinated peptide antigens, RF: rheumatoid factor, SD: standard deviation, N/A, not available. (DOC) [file pone.0020457.s007.doc]

| Sample set | | Number | Female (%) | Age  (mean±SD) | Genotyping Method | Average Success Rate (%) | Autoantibody positivity (%) |
| --- | --- | --- | --- | --- | --- | --- | --- |
| RA | collection1 | 643 | 82.0 | 64.4±12.4 | Human-Hap300,  Human CNV370-Duo | 99.5 | ACPA: 72.3, RF: 86.1 |
|  | collection2 | 327 | 80.0 | 61.3±13.0 | Human610-Quad | 99.8 | ACPA: 82.0, RF: 86.1 |
|  | collection3 | 874 | 85.5 | 62.6±11.6 | Taqman | - | N/A |
|  | collection4 | 1264 | 83.0 | 59.7±11.9 | Taqman | - | N/A |
|  |  |  |  |  |  |  |  |
| Control | collection1 | 934 | N/A | N/A | HumanHap550 | N/A |  |
|  | collection2 | 297 | 68.7 | 58.8±13.2 | HumanHap550 | 99.9 |  |
|  | collection3 | 855 | 39.6 | 38.1±11.9 | Taqman | - |  |
|  | collection4 | 948 | 48.9 | 48.4±16.3 | Taqman | - |  |
