# Supplementary material for: Myelin Basic Protein as a Novel Genetic Risk Factor in Rheumatoid Arthritis—A Genome-Wide Study Combined with Immunological Analyses
Source: PLoS One. 2011 Jun 3;6(6):e20457. doi: 10.1371/journal.pone.0020457 (PMC3108877; doi:10.1371/journal.pone.0020457)
Supplement: Table S2 — Summary of quality control for genome scan results. (DOC) [file pone.0020457.s008.doc]

|  | Collection1 | |  | Collection2 | |
| --- | --- | --- | --- | --- | --- |
|  | Case | Control |  | Case | Control |
| Genotyped samples | 658 | 934 |  | 332 | 302 |
| Excluded |  |  |  |  |  |
| Low call rate | 3 | N/A |  | 0 | 0 |
| High degree of kinship (PI_HAT) | 11 | N/A |  | 1 | 4 |
| Possible contamination | 0 | N/A |  | 0 | 1 |
| Out of Japanese cluster (PCA) | 1 | N/A |  | 4 | 0 |
| Used for statistics | 643 | 934 |  | 327 | 297 |
