# Supplementary material for: Myelin Basic Protein as a Novel Genetic Risk Factor in Rheumatoid Arthritis—A Genome-Wide Study Combined with Immunological Analyses
Source: PLoS One. 2011 Jun 3;6(6):e20457. doi: 10.1371/journal.pone.0020457 (PMC3108877; doi:10.1371/journal.pone.0020457)
Supplement: Table S3 — Association of HLA and PADI4 loci with rheumatoid arthritis in the Japanese population. *risk allele for the disease, **risk allele frequency, and ***p-value in meta-analysis using Cochran-Mantel-Haenszel test. (DOC) [file pone.0020457.s009.doc]

| Chr | dbSNPID | Gene | Allele | DNA Collection | | Genotype counts | | | Success rate | HWE*p* | RAF** | *p*-value | OR | *mhp**** |
| --- | --- | --- | --- | --- | --- | --- | --- | --- | --- | --- | --- | --- | --- | --- |
|  |  |  | Ref.(A1)/Var.(A2) |  |  | A1A1 | A1A2 | A2A2 |  |  |  |  | (95%CI) |  |
| 6p21 | rs2516049 | *HLA-DRB1* | T/C* | 1 | case | 249 | 300 | 94 | 100 | 0.81 | 0.38 | 3.2x10^-21^ | 2.13 |  |
|  |  |  |  |  | control | 558 | 335 | 41 | 100 | 0.3 | 0.22 |  | (1.82-2.49) |  |
|  |  |  |  | 2 | case | 118 | 171 | 37 | 99.7 | 0.033 | 0.38 | 9.5x10^-12^ | 2.35 |  |
|  |  |  |  |  | control | 187 | 99 | 11 | 100 | 0.64 | 0.2 |  | (1.82-3.04) |  |
|  |  |  |  | 3 | case | 367 | 471 | 131 | 99.9 | 0.3 | 0.38 | 3.6x10^-31^ | 2.18 | 5.0x10^-31^ |
|  |  |  |  |  | control | 745 | 434 | 52 | 100 | 0.26 | 0.22 |  | (1.91-2.48) |  |
| 1p36 | rs2240335 | *PADI4* | C*/A | 1 | case | 148 | 313 | 178 | 99.4 | 0.65 | 0.48 | 0.0014 | 1.27 |  |
|  |  |  |  |  | control | 171 | 436 | 324 | 99.7 | 0.25 | 0.42 |  | (1.10-1.46) |  |
|  |  |  |  | 2 | case | 80 | 153 | 93 | 99.7 | 0.28 | 0.48 | 0.0055 | 1.38 |  |
|  |  |  |  |  | control | 47 | 143 | 106 | 99.7 | 0.92 | 0.4 |  | (1.10-1.73) |  |
|  |  |  |  | 3 | case | 228 | 466 | 271 | 99.5 | 0.32 | 0.48 | 3.0x10^-5^ | 1.3 | 2.3x10^-5^ |
|  |  |  |  |  | control | 218 | 579 | 430 | 99.7 | 0.34 | 0.41 |  | (1.15-1.46) |  |
