# Supplementary material for: Myelin Basic Protein as a Novel Genetic Risk Factor in Rheumatoid Arthritis—A Genome-Wide Study Combined with Immunological Analyses
Source: PLoS One. 2011 Jun 3;6(6):e20457. doi: 10.1371/journal.pone.0020457 (PMC3108877; doi:10.1371/journal.pone.0020457)
Supplement: Table S4 — Association of previously reported non- HLA genes in the current study. *p-value in meta-analysis using Cochran-Mantel-Haenszel test. (DOC) [file pone.0020457.s010.doc]

| location | gene | SNP | allele | |  | set1 | | |  | set2 | | | *mhp^*^* |
| --- | --- | --- | --- | --- | --- | --- | --- | --- | --- | --- | --- | --- | --- |
|  |  |  | ref(A1) | var(A2) |  | A1A1 | A1A2 | A2A2 |  | A1A1 | A1A2 | A2A2 |  |
| 6q27 | *CCR6* | rs10946213 | C | T | case | 122 | 329 | 192 | case | 66 | 157 | 103 | 3.9x10^-5^ |
|  |  |  |  |  | control | 235 | 470 | 229 | control | 79 | 133 | 85 |  |
|  |  |  |  |  |  |  |  |  |  |  |  |  |  |
| 6q23 | *TNFAIP3* | rs6931591 | T | C | case | 5 | 87 | 551 | case | 2 | 43 | 281 | 0.0015 |
|  |  |  |  |  | control | 13 | 172 | 749 | control | 2 | 52 | 242 |  |
|  |  |  |  |  |  |  |  |  |  |  |  |  |  |
| 1q23 | *CD244* | rs3766377 | A | G | case | 324 | 251 | 68 | case | 134 | 153 | 39 | 0.012 |
|  |  |  |  |  | control | 389 | 421 | 124 | control | 132 | 126 | 39 |  |
|  |  |  |  |  |  |  |  |  |  |  |  |  |  |
| 2q32 | *STAT4* | rs7574865 | T | G | case | 81 | 310 | 247 | case | 41 | 136 | 149 | 0.14 |
|  |  |  |  |  | control | 96 | 403 | 432 | control | 49 | 128 | 118 |  |
|  |  |  |  |  |  |  |  |  |  |  |  |  |  |
| 9q33-34 | *TRAF1/C5* | rs7035682 | C | T | case | 395 | 218 | 27 | case | 186 | 118 | 22 | 0.20 |
|  |  |  |  |  | control | 546 | 344 | 44 | control | 161 | 118 | 18 |  |
|  |  |  |  |  |  |  |  |  |  |  |  |  |  |
| 1q21-22 | *FCRL3* | rs7522061 | T | C | case | 215 | 322 | 102 | case | 132 | 139 | 55 | 0.27 |
|  |  |  |  |  | control | 343 | 472 | 118 | control | 107 | 140 | 50 |  |
|  |  |  |  |  |  |  |  |  |  |  |  |  |  |
| 5q31 | *SLC22A4* | rs156109 | C | T | case | 24 | 155 | 464 | case | 7 | 80 | 239 | 0.83 |
|  |  |  |  |  | control | 21 | 260 | 653 | control | 8 | 69 | 220 |  |
