# Supplementary material for: Myelin Basic Protein as a Novel Genetic Risk Factor in Rheumatoid Arthritis—A Genome-Wide Study Combined with Immunological Analyses
Source: PLoS One. 2011 Jun 3;6(6):e20457. doi: 10.1371/journal.pone.0020457 (PMC3108877; doi:10.1371/journal.pone.0020457)
Supplement: Table S5 — Replication results of the four regions. The order of SNPs is in accordance with mhp-value. Chromosome and dbSNPID refer to NCBI build 36.3. P-values are calculated using the Cochran-Armitage trend test. *risk allele for the disease, **risk allele frequency, and ***OR, odds ratio with 95% confidence interval. ***p-value in meta-analysis using Cochran-Mantel-Haenszel test. (DOC) [file pone.0020457.s011.doc]

| Chr. | dbSNPID | Gene | Allele | | DNA collection | | Genotype Counts | | | RAF^**^ | *p-*value | OR(95%CI)^***^ | *mhp^****^* |
| --- | --- | --- | --- | --- | --- | --- | --- | --- | --- | --- | --- | --- | --- |
|  |  |  | Ref.(A1) | Var.(A2) |  |  | A1A1 | A1A2 | A2A2 |  |  |  |  |
| 2p11-12 | rs716487 | *CTNNA2* | A | G* | 1 | case | 53 | 226 | 364 | 0.74 | 0.0036 | 1.27(1.08-1.49) |  |
|  |  |  |  |  |  | control | 81 | 410 | 443 | 0.69 |  |  |  |
|  |  |  |  |  | 2 | case | 16 | 132 | 178 | 0.75 | 0.0014 | 1.50(1.18-1.92) |  |
|  |  |  |  |  |  | control | 42 | 114 | 139 | 0.66 |  |  |  |
|  |  |  |  |  | 3 | case | 63 | 362 | 438 | 0.72 | 0.26 | 1.09(0.94-1.26) |  |
|  |  |  |  |  |  | control | 78 | 353 | 417 | 0.70 |  |  |  |
|  |  |  |  |  | pooled | case | 132 | 720 | 980 | 0.73 | 1.4x10^-4^ | 1.21(1.10-1.34) | 1.1x10^-4^ |
|  |  |  |  |  |  | control | 201 | 877 | 999 | 0.69 |  |  |  |
| 10p14 | rs2026628 | none | A | G* | 1 | case | 32 | 230 | 378 | 0.77 | 0.0025 | 1.29(1.09-1.52) |  |
|  |  |  |  |  |  | control | 69 | 379 | 484 | 0.72 |  |  |  |
|  |  |  |  |  | 2 | case | 13 | 101 | 212 | 0.81 | 8.3x10^-4^ | 1.58(1.21-2.05) |  |
|  |  |  |  |  |  | control | 24 | 116 | 157 | 0.72 |  |  |  |
|  |  |  |  |  | 3 | case | 55 | 313 | 494 | 0.75 | 0.39 | 1.07(0.92-1.25) |  |
|  |  |  |  |  |  | control | 57 | 325 | 468 | 0.74 |  |  |  |
|  |  |  |  |  | pooled | case | 100 | 644 | 1084 | 0.77 | 9.3x10^-5^ | 1.23(1.11-1.36) | 1.6x10^-4^ |
|  |  |  |  |  |  | control | 150 | 820 | 1109 | 0.73 |  |  |  |
| 10q21 | rs3910172 | *PLEKHK1* | C* | T | 1 | case | 11 | 129 | 502 | 0.12 | 0.0030 | 1.43(1.13-1.81) |  |
|  |  |  |  |  |  | control | 8 | 143 | 783 | 0.090 |  |  |  |
|  |  |  |  |  | 2 | case | 5 | 75 | 246 | 0.13 | 0.0024 | 1.79(1.22-2.60) |  |
|  |  |  |  |  |  | control | 2 | 42 | 253 | 0.080 |  |  |  |
|  |  |  |  |  | 3 | case | 9 | 173 | 682 | 0.11 | 0.90 | 1.01(0.82-1.26) |  |
|  |  |  |  |  |  | control | 10 | 164 | 669 | 0.11 |  |  |  |
|  |  |  |  |  | pooled | case | 25 | 377 | 1430 | 0.12 | 0.0011 | 1.27(1.10-1.47) | 0.0018 |
|  |  |  |  |  |  | control | 20 | 349 | 1705 | 0.094 |  |  |  |
| 1p31-32 | rs687848 | *MACF1* | A* | G | 1 | case | 364 | 230 | 44 | 0.75 | 0.0030 | 1.28(1.09-1.50) |  |
|  |  |  |  |  |  | control | 461 | 388 | 84 | 0.70 |  |  |  |
|  |  |  |  |  | 2 | case | 179 | 125 | 22 | 0.74 | 0.0039 | 1.44(1.13-1.84) |  |
|  |  |  |  |  |  | control | 135 | 122 | 38 | 0.66 |  |  |  |
|  |  |  |  |  | 3 | case | 449 | 348 | 70 | 0.72 | 0.91 | 1.01(0.87-1.17) |  |
|  |  |  |  |  |  | control | 444 | 332 | 75 | 0.72 |  |  |  |
|  |  |  |  |  | pooled | case | 992 | 703 | 136 | 0.73 | 0.0027 | 1.17(1.06-1.29) | 0.0018 |
|  |  |  |  |  |  | control | 1040 | 842 | 197 | 0.70 |  |  |  |
