# Supplementary material for: Myelin Basic Protein as a Novel Genetic Risk Factor in Rheumatoid Arthritis—A Genome-Wide Study Combined with Immunological Analyses
Source: PLoS One. 2011 Jun 3;6(6):e20457. doi: 10.1371/journal.pone.0020457 (PMC3108877; doi:10.1371/journal.pone.0020457)
Supplement: Table S6 — Oligonucleotide primers used for sequencing of the exons and the promoter region of the MBP gene. (DOC) [file pone.0020457.s012.doc]

| Location | Fragment | Forward Primer | Reverse Primer |
| --- | --- | --- | --- |
| Promoter | P1a | 5'-TCTGTGTGGACACTCTATCC-3' | 5'-TGACAATGAACCCGGGTGCA-3' |
|  | P1b | 5'-TAGTGTTCCAGCTTCCCCAG-3' | 5'-CGGGACTCAGACTTCAGCAC-3' |
|  | P2a | 5'-CAGCTCCCAGAGGAGCAC-3' | 5'-AGGTAAACACAGGCCGATCC-3 |
|  | P2b | 5'-TAAATCCTCCGGGGCTTTGG-3' | 5'-TTCCTGCGAGCCAGGTAAAC-3' |
| Exon1 | E1 | 5'-TTCCGCGTGGGCAGAGCGGG-3' | 5'-TCGGGCCCCGGCGCTGTAAA-3' |
| Exon2 | E2 | 5'-TTGCTTGGAATGGGTGTGGC-3' | 5'-AACAAGGACCAGGCTTCCAC-3' |
| Exon3 | E3 | 5'-ACTATGTGGGCTCACCCTTG-3' | 5'-ACACCATACAGTGAGCCTGG-3' |
| Exon4 | E4a | 5'-TTTACGCTGGGTGCCAAATC-3' | 5'-GTGAGGAAAAGAGGGGGTGA-3' |
|  | E4b | 5'-CGGTGACAGGGGTGCGCCCA-3' | 5'-GCAGCCACAGGTTCTCCACT-3' |
|  | E4c | 5'-TGCGGACTTGATGACTGATG-3' | 5'-GCACTGAAAGAACTAGCCAG-3' |
|  | E4d | 5'-TTCAGGTTGTGACACTTGCC-3' | 5'-ACCCCAGATTTTTAGGGCCA-3' |
|  | E4e | 5'-GCTTGCCCTTTGATGCCAAA-3' | 5'-ATCTTGGCCCTAAGTGCTCA-3' |
|  | E4f | 5'-CCCTAACAACACCCATGCTT-3' | 5'-CCCCTGTGTGTTGCTGCTGC-3' |
|  | E4g | 5'-GACACCTAAGCTGGACATGG-3' | 5'-CAACACAGCACACTGACGTG-3' |
| Exon5 | E5 | 5'-GCACATGCCTTCTTCTCTCC-3' | 5'-CCTGGCTGTCCTCTTCTTTG-3' |
| Exon6 | E6 | 5'-CACCGAGCAATGCACACCAT-3' | 5'-AACACTGCCTTAGGGCTCTC-3' |
| Exon7,8 | E7,8 | 5'-CTCTGGGAGATCTGCATCAT-3' | 5'-CTGGTTGTGTTGGAGGAAGT-3' |
| Exon9 | E9 | 5'-ACTGATTCCTGCTCACCCTG-3' | 5'-AGAGTCAAGGTCAGTCGCCT-3' |
| Exon10 | 10a | 5'-TTCACCCACAGAAGTGCAGC-3' | 5'-GTCACATACCAAAAGCTCCC-3' |
|  | 10b | 5'-ACACCCAATGGCTCAGCATC-3' | 5'-CTGGGAGGAAGTGAATGAGC-3' |
|  | 10c | 5'-CACTAACCCTCCCTGAGAAG-3' | 5'-GTCCAAGACTCCTGGGTTCA-3' |
