# Supplementary material for: Myelin Basic Protein as a Novel Genetic Risk Factor in Rheumatoid Arthritis—A Genome-Wide Study Combined with Immunological Analyses
Source: PLoS One. 2011 Jun 3;6(6):e20457. doi: 10.1371/journal.pone.0020457 (PMC3108877; doi:10.1371/journal.pone.0020457)
Supplement: Table S7 — The list of genetic polymorphisms discovered by sequencing the exons and the promoter region of the MBP gene. *Positions of polymorphisms are according to NCBI Refseq Build 36.3. Polymorphisms are located between position 1 and position 2. (DOC) [file pone.0020457.s013.doc]

| SNP ID | dbSNPID | Allele | | Position1* | Position2* | Freq A1 |
| --- | --- | --- | --- | --- | --- | --- |
|  |  | Ref.  (A1) | Var.  (A2) |  |  |  |
| K1305308 | rs2276276 | C | G | 72819671 | 72819673 | 0.65 |
| K1305307 | rs2276277 | G | A | 72819687 | 72819689 | 0.76 |
| K1305306 | rs470335 | C | T | 72819744 | 72819746 | 0.65 |
| K1305305 |  | G | A | 72819752 | 72819754 | 0.97 |
| K1305304 | rs470493 | G | A | 72819767 | 72819769 | 0.65 |
| K1305303 | rs17026 | A | G | 72819966 | 72819968 | 0.65 |
| K1305302 | rs1049004 | A | G | 72820099 | 72820101 | 0.60 |
| K1305301 |  | C | A | 72820176 | 72820178 | 0.84 |
| K1305300 | rs6565924 | T | C | 72820212 | 72820214 | 0.68 |
| K1305319 |  | C | A | 72820329 | 72820331 | 0.99 |
| K1305299 | rs7229288 | T | A | 72820439 | 72820441 | 0.84 |
| K1335317 |  | G | A | 72821279 | 72821281 | 0.99 |
| K1305298 | rs9199 | T | C | 72821323 | 72821325 | 0.66 |
| K1305297 |  | G | A | 72821353 | 72821355 | 0.97 |
| K1305296 |  | C | T | 72821549 | 72821551 | 0.98 |
| K1305295 | rs12970928 | C | T | 72825513 | 72825515 | 0.71 |
| K1335316 |  | A | G | 72825880 | 72825882 | 0.99 |
| K1305318 | rs12958866 | G | A | 72826394 | 72826396 | 0.96 |
| K1305294 |  | G | A | 72829901 | 72829903 | 0.96 |
| K1335315 |  | C | T | 72830574 | 72830576 | 0.98 |
| K884464 | rs470144 | A | G | 72830629 | 72830631 | 0.68 |
| K1305293 | rs470797 | C | T | 72830971 | 72830973 | 0.68 |
| K1305292 | rs1539991 | G | A | 72850412 | 72850414 | 0.98 |
| K1305291 |  | C | T | 72850490 | 72850492 | 0.89 |
| K1305290 | rs3752070 | C | T | 72850684 | 72850686 | 0.63 |
| K1335308 |  | G | A | 72850993 | 72850995 | 0.84 |
| K671133 | rs12406 | A | G | 72854056 | 72854058 | 0.14 |
| K494641 | rs2282557 | G | A | 72854153 | 72854155 | 0.79 |
| K1305289 |  | A | G | 72854183 | 72854185 | 0.93 |
| K1335314 |  | C | T | 72854503 | 72854505 | 0.99 |
| K1305288 |  | G | A | 72854562 | 72854564 | 0.96 |
| K1305287 |  | C | A | 72854699 | 72854701 | 0.99 |
| K1305286 | rs3752069 | G | A | 72854841 | 72854843 | 0.79 |
| K1335313 |  | G | C | 72855025 | 72855027 | 0.99 |
| K1335306 |  | G | A | 72855201 | 72855203 | 0.99 |
| K1305285 | rs4494640 | C | T | 72855484 | 72855486 | 0.99 |
| K1305284 | rs7236968 | A | T | 72856490 | 72856492 | 0.80 |
| K830726 | rs7232502 | G | A | 72856605 | 72856607 | 0.57 |
| K1335312 |  | C | T | 72856670 | 72856672 | 0.99 |
| K1305317 |  | A | C | 72856836 | 72856838 | 0.99 |
| K1305283 |  | C | G | 72857652 | 72857654 | 0.98 |
| K1305316 |  | A | - | 72857727 | 72857729 | 0.95 |
| K1305281 |  | G | A | 72857810 | 72857812 | 0.99 |
| K1305280 |  | G | A | 72858279 | 72858281 | 0.99 |
| K1335302 |  | G | A | 72906801 | 72906803 | 0.95 |
| K1305279 | rs12327285 | G | A | 72907043 | 72907045 | 0.90 |
| K1305278 |  | T | C | 72907226 | 72907228 | 0.94 |
| K1305277 |  | C | T | 72907232 | 72907234 | 0.94 |
| K772148 | rs7241461 | C | A | 72907502 | 72907504 | 0.43 |
| K1305315 |  | T | C | 72946240 | 72946242 | 0.93 |
| K1305314 | rs731091 | C | A | 72946355 | 72946357 | 0.81 |
| K1305275 |  | C | T | 72973284 | 72973286 | 0.99 |
| K1305274 | rs10454721 | A | G | 72973297 | 72973299 | 0.79 |
| K1305271 |  | C | G | 72973454 | 72973456 | 0.71 |
| K1305270 |  | C | G | 72973586 | 72973588 | 0.73 |
| K1335300 | rs10454722 | C | G | 72973685 | 72973687 | 0.91 |
| K1305269 |  | G | C | 72973741 | 72973743 | 0.97 |
| K1305268 |  | C | G | 72973749 | 72973751 | 0.99 |
| K1335299 |  | G | A | 72973876 | 72973878 | 0.76 |
| K1305313 |  | G | A | 72974050 | 72974052 | 0.89 |
| K1305312 |  | A | C | 72974243 | 72974245 | 0.99 |
| K516234 | rs9966986 | T | C | 72974340 | 72974342 | 0.72 |
| K1305311 |  | A | G | 72974445 | 72974447 | 0.95 |
| K1305310 | rs36069074 | G | C | 72974555 | 72974557 | 0.95 |
| K1305267 |  | C | T | 72974817 | 72974819 | 0.99 |
| K1305266 |  | TTTTA | - | 72975101 | 72975107 | 0.18 |
| K991863 | rs1789094 | G | A | 72975172 | 72975174 | 0.76 |
| K1305264 | rs4890901 | T | C | 72975342 | 72975344 | 0.79 |
| K1305309 |  | - | ATGA | 72975350 | 72975351 | 0.99 |
