# Supplementary material for: Myelin Basic Protein as a Novel Genetic Risk Factor in Rheumatoid Arthritis—A Genome-Wide Study Combined with Immunological Analyses
Source: PLoS One. 2011 Jun 3;6(6):e20457. doi: 10.1371/journal.pone.0020457 (PMC3108877; doi:10.1371/journal.pone.0020457)
Supplement: Method S1 — Sequencing of the exons and the promoter region of the MBP gene. (DOC) [file pone.0020457.s014.doc]

***Sequencing of the exons and the promoter region of the MBP gene***.

A total of 84 DNA samples of healthy Japanese population controls were used. Conditions for PCR were as follows: denaturation at 94°C for 10mins, followed by 40 cycles of 94°C for 30s, 55-64°C for 30s, 72°C, for 1min; and a final extension step of 72°C for 10mins. Sequencing was performed as described[1] using Dye Terminator technology with ABI PRISM 3730 DNA Analyzer (Applied Biosystems, Foster City, CA, USA). Polymorphisms in sequenced regions were identified with *Genalys* software[2].

References

1. Vasilescu A, Heath SC, Ivanova R, Hendel H, Do H, et al. (2003) Genomic analysis of Th1-Th2 cytokine genes in an AIDS cohort: identification of IL4 and IL10 haplotypes associated with the disease progression. Genes Immun 4: 441-449.

2. Takahashi M, Matsuda F, Margetic N, Lathrop M (2003) Automated identification of single nucleotide polymorphisms from sequencing data. J Bioinform Comput Biol 1: 253-265.
