# Supplementary material for: Myelin Basic Protein as a Novel Genetic Risk Factor in Rheumatoid Arthritis—A Genome-Wide Study Combined with Immunological Analyses
Source: PLoS One. 2011 Jun 3;6(6):e20457. doi: 10.1371/journal.pone.0020457 (PMC3108877; doi:10.1371/journal.pone.0020457)
Supplement: Method S2 — Bioinformatics analysis. (DOC) [file pone.0020457.s015.doc]

***Bioinformatics analysis***

Genome sequence alignment of 14 placental mammals was obtained from the UCSC genome browser (http://genome.ucsc.edu). Motif search was carried out by the Jaspar database ([http://jaspar.cgb.ki.se](http://jaspar.cgb.ki.se/)) using ‘Jasper Core Subset’ which contains 138 matrices for known *cis*-acting elements. The matrices were converted into bit scores and used to search against the genomic sequences around the SNP of interest.
