# Supplementary material for: Myelin Basic Protein as a Novel Genetic Risk Factor in Rheumatoid Arthritis—A Genome-Wide Study Combined with Immunological Analyses
Source: PLoS One. 2011 Jun 3;6(6):e20457. doi: 10.1371/journal.pone.0020457 (PMC3108877; doi:10.1371/journal.pone.0020457)
Supplement: Method S3 — Immunoblotting of anti-MBP antibody. (DOC) [file pone.0020457.s016.doc]

***Immunoblotting of anti-MBP antibody***

0.5µg of MBP derived from human brain was separated in a BisTris SDS-polyacrylamide gel (Invitrogen, Carlsbad, California, US) and transferred to nitrocellulose membrane (GE Healthcare Bio-sciences, Uppsala, Sweden). After treatment with SuperBlock Blocking Buffer (Thermo Fisher Scientific, Waltham, MA) for 10 minutes, blots were incubated overnight at 4°C with rabbit polyclonal anti-MBP antibody (Thermo Fisher Scientific, Waltham, MA) as positive control, or with human plasma (1:750 in 5% milk powder in TBST). The strips were then incubated with alkaline phosphatase conjugated anti-rabbit IgG or anti-human IgG (1:7500) for 1.5 hours at room temperature, followed by detection with NBT (Sigma, St. Louis, MO) and BCIP (Sigma, St. Louis, MO).
