# Supplementary material for: Myelin Basic Protein as a Novel Genetic Risk Factor in Rheumatoid Arthritis—A Genome-Wide Study Combined with Immunological Analyses
Source: PLoS One. 2011 Jun 3;6(6):e20457. doi: 10.1371/journal.pone.0020457 (PMC3108877; doi:10.1371/journal.pone.0020457)
Supplement: Method S4 — Evaluation of non-specific binding of secondary antibodies. (DOC) [file pone.0020457.s017.doc]

***Evaluation of non-specific binding of secondary antibodies***

Human purified IgM (MP Biomedicals LLC, Santa Ana, CA) at 5ng/µl and serial dilution of human purified IgG (R&D systems, Minneapolis, MN) were coated in duplicate and incubated overnight. After washing and blocking, rabbit polyclonal anti-human IgG was applied to each well and OD value was measured at 30min. Cross reactivity of IgM with rabbit polyclonal anti-human IgG antibody was estimated under standard curve of the serial dilution.
