# Supplementary material for: Myelin Basic Protein as a Novel Genetic Risk Factor in Rheumatoid Arthritis—A Genome-Wide Study Combined with Immunological Analyses
Source: PLoS One. 2011 Jun 3;6(6):e20457. doi: 10.1371/journal.pone.0020457 (PMC3108877; doi:10.1371/journal.pone.0020457)
Supplement: Method S5 — Amino acid analysis. (DOC) [file pone.0020457.s018.doc]

***Amino acid analysis***

Amino acid analysis was performed as a standard laboratory test by SRL (Tachikawa, Tokyo, Japan) for detection of citrullination level in MBP derived from human brain used in ELISA.
